# Enclosing the commons: reasons for the adoption and adaptation of enclosures in the arid and semi-arid rangelands of Chepareria, Kenya

**DOI:** 10.1186/s40064-015-1390-z

**Published:** 2015-10-13

**Authors:** John N. Wairore, Stephen M. Mureithi, Oliver V. Wasonga, Gert Nyberg

**Affiliations:** Department of Land Resource Management and Agricultural Technology (LARMAT), University of Nairobi, P.O. Box 29053-00625, Nairobi, Kenya; Triple L Research Initiative, Uppsala, Sweden; Department of Forest Ecology and Management, Swedish University of Agricultural Sciences (SLU), 901 83 Umea, Sweden

**Keywords:** Communal land, Arid and semi-arid lands, Rangeland enclosures, Rehabilitation, West Pokot County

## Abstract

The adoption and adaptation of enclosures in the arid and semi-arid rangelands of sub-Saharan Africa is driven and sustained by a combination of factors. However, reviews indicate that these factors cannot be generalized, as they tend to be case specific. A study was therefore conducted to explore the history and reasons for enclosure establishment in Chepareria, a formerly degraded communal rangeland in north-western Kenya. While Vi-Agroforestry Organization accounting for 52.5 % was the main source of knowledge on enclosure establishment; it has now emerged that rangeland enclosures among the Pokot pastoral community existed prior to land management interventions by Vi- Agroforestry. Results indicated that there are three categories of enclosures which were established for boundary demarcation, provide grazing reserves, enable proper land management, facilitate crop cultivation in a pastoral setup and to curb land degradation. The role of self-trigger [accounting for most of the spontaneous enclosures (73.5 %)] indicates the continued establishment and expansion of areas under enclosure management as private land ownership accounting for 51.7 % of enclosure tenure continues to gain momentum in Chepareria. While rangeland enclosures in Chepareria were mainly established for boundary demarcation, to alleviate pasture scarcity and enable proper management of formerly degraded areas; they have facilitated land restoration and rehabilitation by increasing flexibility in land, fodder and livestock management amongst agro-pastoralists in Chepareria over the last three decades. To ensure that rehabilitated areas do not revert to their previously degraded state; technical interventions are needed to allow for a more intensive use of rangeland resources within enclosed areas.

## Background

Most rangelands are caught in a spiral of desertification, land degradation and drought (DLDD), deforestation and land fragmentation (FAO 2010). DLDD has been identified as key threat to both dryland and non-dryland communities, and sustainable economic development in drylands, particularly in developing nations (UNCCD 2012, 2013), as they lead to reduced human well-being due to increased poverty and vulnerability of the affected dryland populations (MA 2005). Land degradation, in particular has led to increased food insecurity; compromised the ecosystem integrity and consequently lowered the quality of life of most dryland communities (Eswaran et al. 2001; MA 2005; Reynolds et al. 2007).

Many attempts to rehabilitate degraded rangelands have failed (Meyerhoff 1991; de Groot et al. 1992; Wasonga 2009; Mureithi et al. 2010) as they placed more importance on the physicality and technicality of the interventions than the socio-economic and cultural needs of the people (Mureithi et al. 2010). Consequently, there have been increasing calls for holistic, multidisciplinary and integrated ecosystem approaches when rehabilitating fragile ecosystems (Harris et al. 1996; UNDP/UNCCD/UNEP 2009). Rehabilitation of degraded rangelands by enclosing the commons -enclosures- is a successful local approach in combating land degradation in rangelands and is gaining prominence (Verdoodt et al. 2010).

Enclosures refer to areas closed off from grazing for a specified duration of time in order to allow the regeneration of vegetation (Behnke 1986). Studies in Somalia (Gaani 2002), Tanzania (Mwilawa et al. 2008), China (Bauer 2005), Sudan (Behnke 1985, 1986; Nedessa et al. 2005), Ethiopia (Mengistu et al. 2005; Mekuria et al. 2007; Keene 2008; Beyene 2009) and in Kenya (Meyerhoff 1991; Makokha et al. 1999; Mureithi et al. 2010, 2015; Wasonga 2009; Opiyo et al. 2011; Kigomo and Muturi 2013; Wernersson 2013; Svanlund 2014) all illustrate that rangeland enclosure is indeed, a well-known and successful management tool for the restoration of degraded rangelands within and beyond East Africa.

In Chepareria, a formerly communal and degraded ward in West Pokot County, enclosures were mainly established to address pasture shortage. Enclosures as a land management approach enabled individuals to properly manage land, fodder and livestock hence creating stable environment for the local pastoral community in Chepareria (Wairore et al. 2015a). Through increased flexibility in land use, pasture and livestock management, private enclosure owners in Chepareria have not only been able to restore degraded lands but also adopt alternative income generating activities (IGAs). These have resulted in improved standards of living amongst agro-pastoralists in Chepareria ward (Makokha et al. 1999).

While enclosures have been able to foster rangeland restoration and rehabilitation, it is now emerging that they were not specifically established for land rehabilitation, particularly in Chepareria. As a land use fragmentation/management approach, we hypothesize that enclosures were established for diverse reasons, particularly if their categories/types, time of establishment and source of information/knowledge on how to establish them vary. By drawing inference from Chepareria, this study sought to document the history of enclosures in Chepareria, sources of information/knowledge on enclosure establishment and explore the reasons for the enclosure movement in the formerly degraded rangelands. More importantly, we seek to identify how land use fragmentation/management through rangeland enclosures has shifted risks of degradation from previously communal rangelands to private allotments in enclosed areas. Understanding these key thematic areas is important in the upscaling of enclosures to other similar rangelands within and beyond East Africa.

## Methods

### Study area

**Fig. 1:**
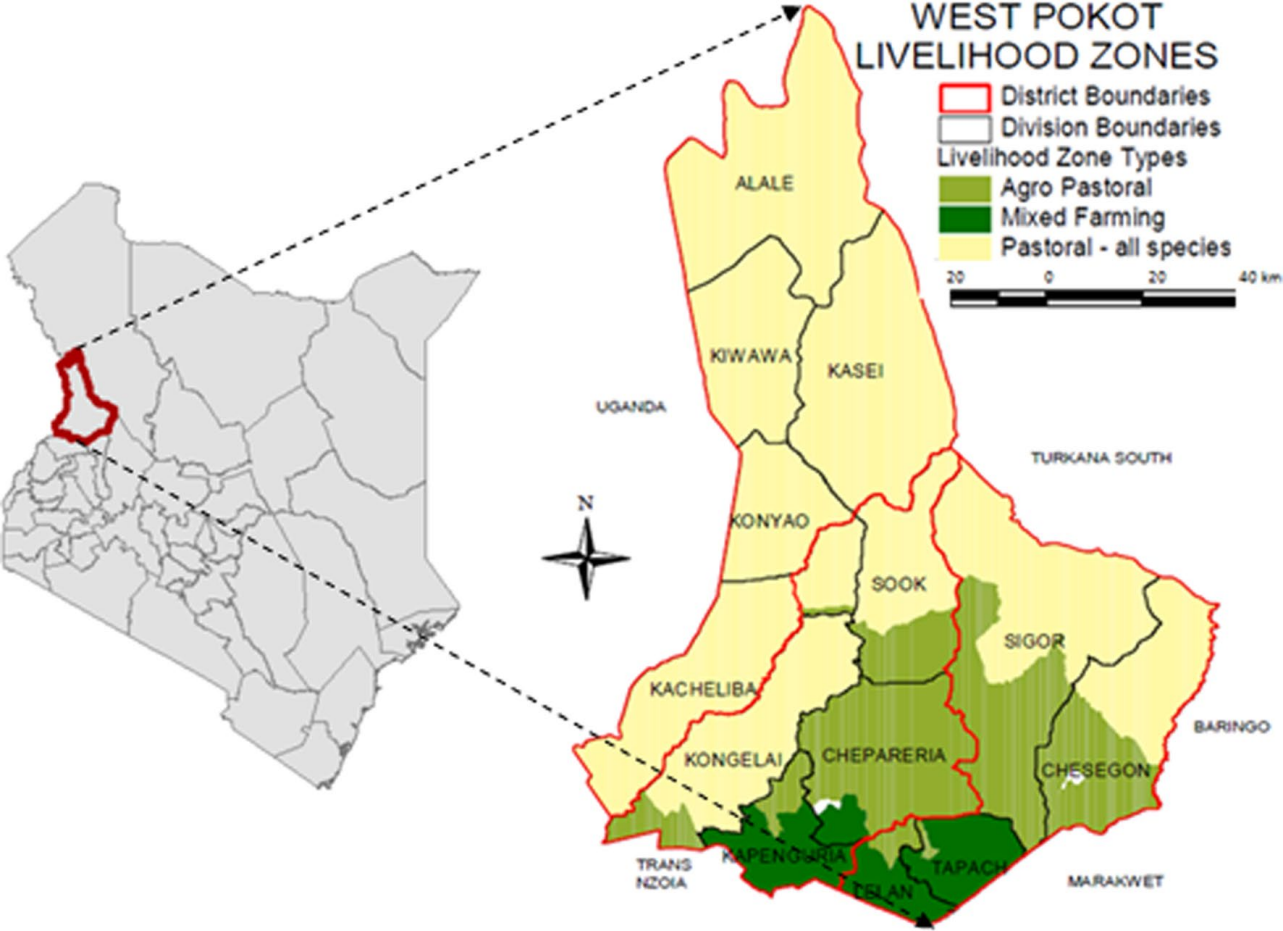
Location of West Pokot County in Kenya and *inset* Chepareria Ward in West Pokot County

The study was conducted in Chepareria ward in West Pokot County (Fig. 1). The ward lies between latitude 1°15′ to 1°55′N and longitude 35°7′ to 35°27′E. The region experiences a highly variable and seasonal climate as is the case with similar arid and semi-arid lands (ASALs) in Kenya. While rainfall in Chepareria increases with increasing altitude, it averages 600 mm (County Government of West Pokot 2013) and is bimodal with the long rains between March and May (MAM) and the short rainy period from August to November as described by the National Drought Management Authority (NDMA 2014). The average annual temperature in Chepareria ranges from 24 to 38 °C (County Government of West Pokot 2013).

Soils vary considerably from shallow and friable in the lowlands to deep and well-drained in the upper areas (Sposito 2013). In terms of fertility, it varies from moderate to low fertility as described by FAO (2006). The vegetation is steppe-like, though grasslands interspersed with native and exotic tree species dominate. The region is mainly inhabited by the Pokot tribe; a community with a long history of livestock keeping in Kenya. According to the Kenya National Bureau of Statistics (KNBS), Chepareria ward has a population of about 41,563 persons (KNBS 2009).

### Sampling design and data collection

Ywalateke, Chepkopegh and Morpus locations were purposively selected for this study. The three locations represent the areas where the Non-Governmental Organization (NGO) Vi-Agroforestry (Vi-AF) conducted intensive extension on agroforestry and enclosure establishment in Chepareria. Using a checklist of more than 400 enclosure owners in each location, systematic random sampling was used to select a sample of 40 enclosure owners in each location, giving a total sample of 120 households.

A combination of data collection instruments were used in this study. A semi-structured questionnaire was used to collect data on household demographics, characteristics of selected enclosures, history, sources of information/knowledge and reasons for rangeland enclosure establishment. Five key informant interviews (KIIs) and eight focus group discussions (FGDs) were also conducted to clarify and obtain further information on responses that appeared unclear and compliment information gathered through the semi-structured questionnaire, particularly on the reasons for enclosure establishment and how they have enabled respondents address land degradation in the area. To contextualize this study, extensive literature review was also conducted to identify and relate our findings on reasons and implications of rangeland enclosures on land restoration and rehabilitation.

### Data analysis

Qualitative data gathered from literature review, FGDs, and KIIs was compiled, organized and consolidated using summary tables into different topics addressed during this study. This information was used to help interpret and clarify qualitative data collected from household interviews. The statistical package for the social sciences (SPSS) was used to analyze data collected from semi-structured questionnaires. Descriptive statistics such as means, standard deviation (SD) and percentages were used to present results on the history, categories and reasons for enclosure establishment in Chepareria. Bivariate correlation was used to determine factors influencing the choice of enclosure categories amongst enclosure owners in Chepareria. Significant correlations were detected using Pearson’s coefficient two-tailed test of significance. Information obtained from literature reviewer was not only essential in contextualizing the study but also helped in relating our results and findings to those of previous studies.

## Results

### Selected demographic and enclosure characteristics

Majority of the households interviewed were headed by males (73.3 %), most of whom (42.5 %) had an average age of between 36 and 50 years. While a majority of the respondents were married (97.5 %), the 2.5 % of those who are not married corresponds to the 0.8 % of household headed by respondents below 20 years as indicated in Table 1. While a significant 56.3 % of respondents have attained primary education; there remains a considerable 29.4 % of household heads who have not accessed education. There was a significant negative correlation between education level attained and age of household head (p ≤ 0.01) indicating a trend of increased access to education among younger household heads compared to their older counterparts. Though weak, the observed significant negative correlation between education level attained and gender of the household head (p ≤ 0.05) indicates that access to education among women is still an issue in Chepareria. In Chepareria, most households have an average family size of 7 ± 3. The observed significant positive correlation between family size and age of household head (p ≤ 0.05) indicates that older respondents are likely to have a larger family size compared to younger household heads. This relationship can be associated with the observed significant negative correlation between the age and education level attained by the household head. Enclosures averaged 5.01 (±4.38) ha with an increasing trend towards formalization of land tenure as indicated by the 51.7 % of enclosures under private ownership.

**Table 1:** Selected demographic and enclosure characteristics of sampled households in Chepareria

	%	Mean (SD)
Household head		
Gender		
Male	73.3	
Female	26.7	
Age (years)		
0–20	0.8	
21–35	36.7	
36–50	42.5	
50+	20.0	
Marital status		
Single	2.5	
Married	97.5	
Education level attained		
None	29.4	
Primary	56.3	
Secondary	8.4	
Post-secondary	5.9	
Average family size (SD)		7 (3)
Enclosure		
Average enclosure area (SD)		
ha		5.01 (4.38)
Enclosure tenure		
Private	51.7	
Communal	48.3	

### History, categories of enclosures and sources of knowledge on enclosure establishment in Chepareria

In order to understand how individuals gain access to the land to enclose, the aged respondents indicated that enclosures existed even before the colonial period. Due to their migratory nature, these enclosures would be abandoned and new ones established in the next settlement area. During the colonial era, grazing regulations which partitioned the Pokot grazing lands into sections were instituted by the administrators. Later, these areas were divided into group ranches under the group ranch management system in a bid to control livestock diseases. Owing to their migratory lifestyle, the Pokots were not in favour of this management system. After Kenya gained her independence in 1963, the instituted group ranch committees were not able to regulate grazing like during the colonial times and the scheme was poorly coordinated hence overstocking and land degradation. Since most individuals were not satisfied with the group ranch operations, the land enclosure movement easily received support of group ranch committee members, especially after witnessing the initial results of the project in demonstration sites set in schools and churches. This was followed by community discussions around 1990–1993 which sought to strengthen the resolution of group ranch members to demarcate the group ranches into individual land parcels. However, this did not happen until 1997, when several group ranches passed a resolution to wind up group ranches in favour of individual land holdings. Informal group ranch subdivisions in Chepareria were hastily conducted and completed. Through these subdivisions, individuals were given rights to use their land holding which represented some *de facto* degree of ownership. As of today, the process of adjudication is still on-going. While there exists legal technicality of survey and registration of individual title deeds among group ranch members; there is proof that this is happening as evidenced by the 51.7 % of respondents who already have titled deeds as indicated in Table 1.

Most of the enclosures were established after technical interventions in land management by Vi-AF which started in 1987 as evidenced by 89.2 % of the sampled enclosures which were established in the last 30 years (Table 2). However, 10.8 % of the enclosures were established prior to Vi-AF land management intervention in 1987 as indicated in Table 2. The age of enclosure (years since effective protection) was significantly correlated to the age of household head (p ≤ 0.01), and in turn influenced the category and acreage (ha) of enclosures (p ≤ 0.01) established in Chepareria.

**Table 2:** Age distribution of enclosures establishment in Chepareria

	Count	%
Enclosure age		
<10	45	37.5
11–20	42	35.0
21–30	20	16.7
31+	13	10.8
Total	120	100.0

**Fig. 2:**
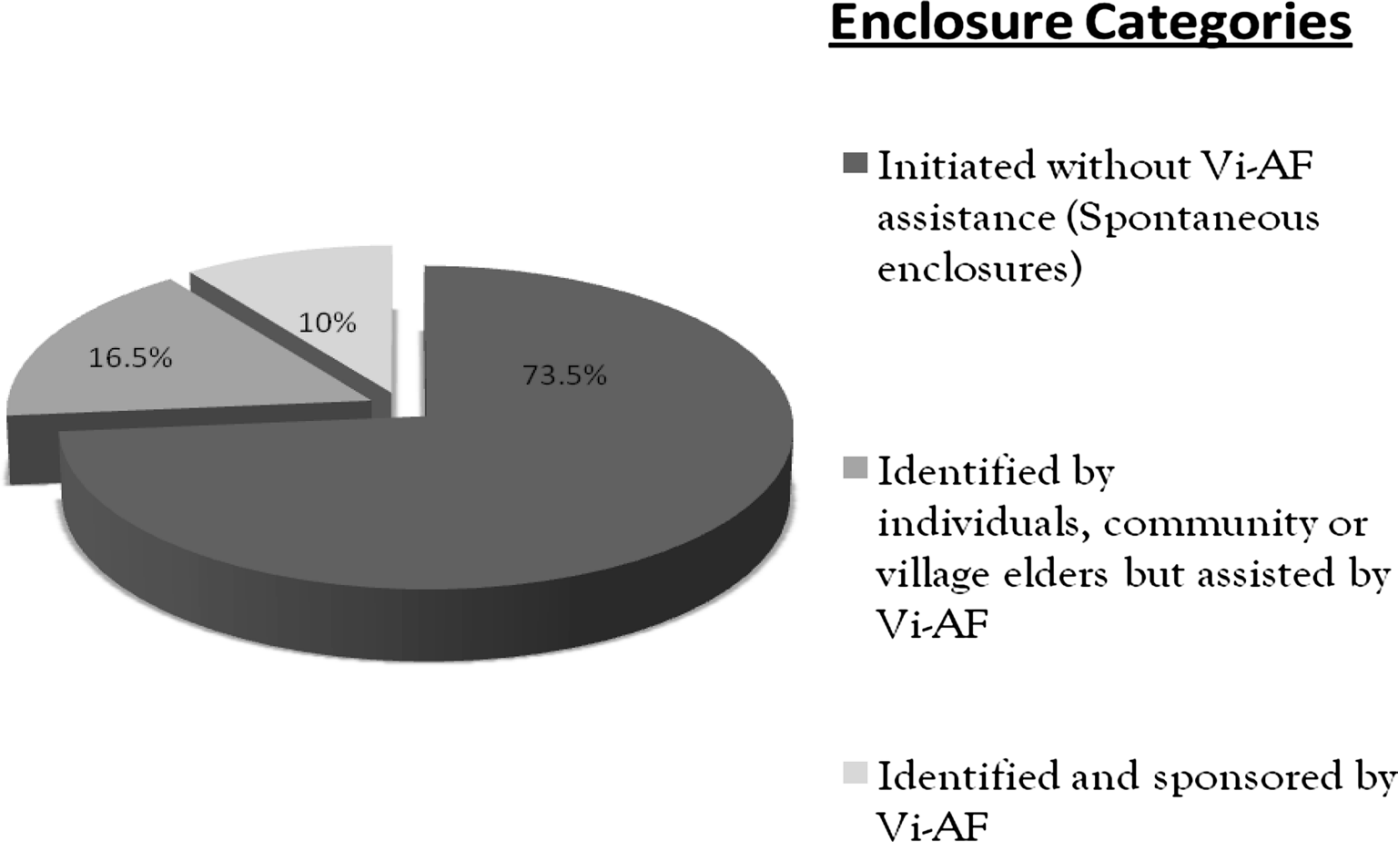
Categories of rangeland enclosures in Chepareria

There exist three categories of enclosures in Chepareria namely: Enclosures identified and sponsored by Vi-AF (10 %); Enclosures identified by farmers, elders or the community but assisted by Vi-AF (16.5 %) and Enclosures initiated without Vi-AF assistance-spontaneous enclosures (73.5 %) as indicated in Fig. 2. The existing significant negative correlation between enclosure category and age of enclosure and household head (p ≤ 0.01) indicates a trend of increasing establishment of spontaneous enclosures, particularly among the younger generation over recent years.

Vi-AF was the main source of knowledge and information on enclosure establishment as indicated by (52.5 %) of respondents who benefitted from the Vi-Tree Planting Project (Vi-TPP). Neighbours/community (27.5 %), local leaders (22.5 %) and parents (15.8 %) were other common techniques of knowledge and information sharing on enclosure establishment in Chepareria. Other included field visits, government extension officers and other NGOs accounting for 2.5, 2.5 and 0.8 % respectively as indicated in Table 3.

**Table 3:** Sources of information on enclosure establishment and management in Chepareria

	Responses	N = 120	%
Sources of information/knowledge on how to establish enclosures	Vi Agroforestry	63	52.5
	Neighbours/community members	33	27.5
	Local leaders	27	22.5
	Parents	19	15.8
	Government extension officers	3	2.5
	Field visits	3	2.5
	Other NGOs	1	0.8

### Reasons for enclosure establishment and sources of enclosure information

Results indicated that enclosures were mainly established for boundary demarcation owing to underlying tenure insecurity, preserve and properly manage livestock pasture and in order to properly manage land at 70.8, 65.0 and 52.5 % respectively. With increasing adoption of agriculture, enclosures were also established to facilitate crop production (31.7 %) either for subsistence or for sale. Being a previously degraded area; enclosures were also established to curb land degradation (26.7 %) and gain diverse environmental/ecosystem benefits and services (14.2 %) as indicated in Table 4.

**Table 4:** Reasons for enclosure establishment in Chepareria

Responses	N = 120	%
Boundary demarcation/tenure insecurity	85	70.8
Preserve pasture	78	65.0
Proper land management	63	52.5
Enable crop production	38	31.7
Curb land degradation	32	26.7
Gain environmental benefits	17	14.2

## Discussion

### History of enclosures in Chepareria

Rangeland enclosures in Chepareria existed even before interventions by Vi-AF through their Vi-TPP project which involved intensive extension on enclosure establishment and agroforestry. Our results indicated that although Vi-AF started operations in 1987, enclosures in Chepareria were established as early as 1967. This feature is supported by the fact that there are more than 10.8 % of enclosures which were established before 1987 as indicated in Table 2. Similar results have been reported by Makokha et al. (1999) who observed that the Pokot people were using customary enclosures before the Vi-AF Project. According to Makokha et al. (1999) customary enclosures were mainly used for calves, milk cows and sick animals and for the cultivation of millet and sorghum, and these small areas were mainly enclosed with thorny branches. Due to their migratory lifestyle, these enclosures would be abandoned and new ones established in their next settlement area. Makokha et al. (1999) recounts that the introduction of the group ranch management systems by the colonial administration changed the Pokot way of life (traditional system); in its place, this system confined animals to restricted areas in the name of disease control (Nangulu 2009). Previous studies indicated that this system was poorly coordinated (management), eventually leading to overstocking, overgrazing and land degradation (Makokha et al. 1999). It is then that Vi-AF through their Vi-TPP project started conducting intensive extension on enclosure establishment using demonstration sites in schools and churches, with an aim to address pasture scarcity and create stable environments for the local pastoral community (Kitalyi et al. 2002). Under the project, individuals were encouraged to use live-fences to control stocking density and enhance rotational grazing, plant trees and carry out grass reseeding (Svanlund 2014). Initially, the project worked in churches and schools which acted as demonstration plots. Once the project obtained the go-ahead from its members, the most degraded areas were selected and rehabilitation measures established. Originally, the Vi-TPP worked on a top-down approach during the introductory years. However, successful rehabilitation of the demonstration plots saw more individuals interested in enclosing their land hence a transition into a demand-driven scenario (Makokha et al. 1999).

The evolution of Vi-AF interventions towards land improvement through the establishment of pasture enclosures consisted of initially working on public land in schools and churches which after 3 years acted as demonstration plots for the local community. The transition from the establishment of demonstration plots on public land to the establishment of spontaneous enclosures is estimated to have taken about 7 years (1987–1994). Thereafter, Vi-AF conducted intensive extension on enclosures between 1995 and 2001 before phasing out the project in 2002. Enclosure categories were found to be significantly correlated to the age of household head (p ≤ 0.01) with older households having older enclosures and which are likely to have been established or supported by Vi-AF.

### Enclosure categories

Enclosures identified and sponsored by the project were established using the top-down approach due to the fact that the local community did not have confidence in the project and the results of the project were not definite. Similar results were reported by Makokha et al. (1999) who observes that under this enclosure category, a Plantation Management Committee (PMC) consisting of community members, local administration and project staff was constituted to ensure that the community was adequately informed of the project activities and activities were undertaken to the latter. Therefore, this enclosure category only accounts for 10.0 % (Fig. 2) of enclosures in Chepareria as they served to introduce and convince the community that the technique being proposed was effective and worthwhile to adopt.

Enclosures identified by individuals, elders or the community but assisted by the project were established where an elder in the village or any other member of the community approached the project for assistance. If the request was accepted, the project would convene a *baraza* in which its role in the project would be discussed. Under this engagement, the community members would fence off the stipulated land while the project would hire casual labourers to prepare micro catchments, plant tree seedlings and grass seeds. According to Makokha et al. (1999), individual owners of these lands were expected to take care of the enclosures for a minimum of 3 years before allowing animals into the enclosure.

Enclosures initiated without Vi-AF assistance (also referred to as spontaneous enclosures) were established after individuals witnessed successful rehabilitation of degraded lands in demonstration plots and improved enclosures in their locality. Previous studies in Chepareria have reported that Labour needs were met by family members or neighbours under the *sikom*—Pokot communal labour system in which the community assists one of its own to undertake a specific task which requires more labour than the family can provide-system (Makokha et al. 1999). Fencing in this enclosure category was mainly achieved using dead branches of *Acacia nilotica* although a few individuals planted live fences using sisal or euphorbia during the wet season. It has been observed in previous studies that the transition from the establishment of enclosures in demonstration plots to the spontaneous enclosures took roughly 7 years after which Vi-AF was less active in the area (Makokha et al. 1999). Therefore, this category accounts for over half of the enclosure types in Chepareria (73.5 %) given that most enclosures were established after this period. Besides in Chepareria, the establishment of spontaneous enclosures has also been found to be on the increase in Lake Baringo Basin as described in previous studies by Mureithi et al. (2010) in Baringo County, Kenya. Overall, spontaneous enclosures indicated continued establishment of rangeland enclosures in the formerly degraded rangelands as individuals seek to tap on the various benefits derived from rehabilitated rangelands in private or communal enclosures.

### Sources of information on enclosure establishment

The existence of enclosures in Chepareria as early as 1967 reinforces observations by Makokha et al. (1999) describing that enclosures were being used before the Vi-AF Project. Vi-AF through their intensive extension on agroforestry and enclosure establishment accounted for 52.5 % and was the main source of knowledge and information on enclosure establishment in Chepareria (Table 3). Through observation or association with the project, individuals gained knowledge on how to establish rangeland enclosures and manage them as a land management approach. Individuals also learnt how to establish enclosures by adopting what their neighbours were doing. Many of those who were not convinced by the Vi-TPP would later establish enclosures after witnessing the transformative ecological change within enclosed areas. These households hugely relied on the advice of their neighbours and community members when enclosing their individual farms. The role of local leaders and local level administrators is significant in not only spreading information but also advising community members within their jurisdiction on how to enclose degraded areas. This is very crucial given that it’s the local leaders who were charged with the role of land demarcation and also served in the land committees. Parents, as custodians of knowledge on enclosure establishment accounted for 15.8 % of the various sources of knowledge/information. By training a new generation of enclosure owners and managers, parents have passed on knowledge on enclosure establishment and management to their children either through hands-on involvement or casual observation. When their children inherit land, they are then able to use this knowledge when establishing their own enclosures. Other enclosure owners acquired knowledge from government extension officers, attending field or farm visits in other areas and through other NGOs as indicated in Table 3.

### Reasons for enclosure establishment in Chepareria

There are combinations of factors which are attributable to the establishment of rangeland enclosures in sub-Saharan Africa (SSA). Previous studies by Forester (2002) and Behnke (1986) in Ethiopia and Sudan respectively have shown that there are diverse objectives for the establishment of rangeland enclosures in drylands. Our findings in Chepareria rangelands indicated that enclosures were established for:

#### Boundary demarcation

The enclosure movement in Chepareria was initiated by pastoralists to address pasture scarcity in the area and create stable environments for the local pastoral community. Similar results have been reported by Graham (1988) who observed that enclosures in East African rangelands are in some instances, initiated by pastoralists owing to the perception that good land is becoming scarce. Increased land degradation in Chepareria not only reduced the available good land but also increased pasture scarcity among the Pokot pastoral community in Chepareria.

While studies by Graham (1988) and McCarthy et al. (2003) have reported that rangeland enclosures in SSA are prevalent where privatization supported by the state or planners is believed to encourage a more responsible and rational use of the rangelands; we reiterate that the establishment of enclosures in Chepareria was driven the local pastoral community. In Chepareria, policies favouring the group ranch management system were highly disliked by the community; particularly after the exit of colonialists as the group ranch system was poorly coordinated hence leading to overgrazing and land degradation as cited by Makokha et al. (1999). With increasing evidence of the restorative success of rangeland enclosures within the demonstration sites, enclosures were increasingly established in order to lay claim to a demarcated area hence grazing rights. Similar findings were reported by Graham (1988). The winding up of group ranch management in favour of individual landholdings created the impetus for increasing establishment of rangeland enclosures as a form of land ownership in Chepareria. According to Makokha et al. (1999) individual landholdings created some degree of land independence and ownership of enclosed areas in Chepareria. Similar results have been reported by Saxer (2014). Our studies found that the observed success of rangeland enclosures in addressing pasture scarcity, restricted access to enclosed areas and a reduction of the available communal land, increasing establishment of enclosures to own land was also driven by the fact that the largest share of people were putting up fences because other people were putting up fences. Chances that those who did not enclose land would be left out in communal lands easily accessible by others or get the poor lands owing to allocation bias informed by the spontaneous establishment of enclosures for boundary demarcation and land ownership.

Increasing tenure insecurity owing to spontaneous enclosure establishment, restricted access to enclosed areas and a shrinking resource base for pastoralists (communal land) saw more individuals interested in securing and managing private grazing and farming areas for various household needs. This could only be feasible if individuals had some form of *de facto* rights on the land hence the need for clarity on boundaries. In a previous study in Chepareria, Makokha et al. (1999) observed that the recognition of group ranch representatives as owners of the land as provided under Section 287 of the Land (Group Representative) Act (Kenyalaw.org 2012) allowed for all members of a group ranch to have an equal and undivided share of the ranch and any other group resource. It is against this background that private enclosures were developed and are still being developed as some land is still held under the group ranch/communal tenure regime in Chepareria as indicated in Table 1.

#### Pasture preservation

The Pokot community being a predominant pastoral community, rangeland enclosures in Chepareria were mainly established to address pasture scarcity in the area. The establishment of enclosures was seen a viable approach to enhance land management and create stable environments for the local pastoral community. Similar results have been reported by Makokha et al. (1999) who observed that pasture enclosures were established in order to provide grazing reserves during the dry season as communal grazing and livestock migration decreased. More so, similar findings were observed in Chepareria by Wairore et al. (2015b) who observed that rangeland enclosures in Chepareria have fostered increased flexibility in land use, fodder and livestock management hence enabling individuals to control grazing throughout the year. Previous findings by Desta et al. (2013) and Wairore et al. (2015a) in Ethiopia and Kenya respectively have reported that through various enclosure management regimes, individuals are able to maximize on land use, ensure flexibility and provide fall-back options in the face of climate change impacts such as drought. In the Cantabrian Mountains of Spain, similar results have been reported by Álvarez-Martínez et al. (2013) who observed that through increased flexibility in land, fodder and livestock management, rangeland enclosures are increasingly being used to manage livestock and control biomass.

Using enclosures, individuals in Chepareria have been able to preserve natural pasture within their fields for dry season grazing. In the event that this reserve pasture is not required, individuals can choose to cut-and-carry this fodder and store it as hay. Similar findings have been observed in Ethiopia by Kindeya (Desta et al. 2013) who observed that the grazing reserves or protected pasture enables individuals to maintain livestock productivity during the dry season. On the other hand, those with large enclosures also allow others, particularly those with small enclosed areas and large herds to graze in their fields at a fee in what is commonly termed as contractual grazing. Previous studies in Kenya and Ethiopia have reported contractual grazing as common practice amongst enclosure owners in East Africa (Makokha et al. 1999; Keene 2008; Beyene 2006, 2011; Mureithi et al. 2015), one which would not be possible if the rangelands were still held communally (Keene 2008; Beyene 2010).

Besides natural pasture, artificial reseeding involving the cultivation of high-yielding grass varieties such as *Chloris gayana* was also prominent, particularly in the wetter regions of Chepareria. Fodder production enables enclosure owners and by extension other community members to cope with drought since excess fodder can always be sold to those in need. The grass can also be cut and stored as hay and used as fodder in case of drought. More so, crop residues are rarely sold but are stored to be used during the dry season or even drought. Previous studies in Ethiopia by Abule et al. (2005), Kamara et al. (2004) and Desta et al. (2013) have observed that the preserved pasture or fodder also provides strategic grazing fields for the lactating stock during the dry season, the young stock or is used for fattening bulls.

#### Proper land management

The establishment of enclosures in Chepareria was also observed to be due to an inherent need to manage and utilize land as individuals saw fit. Increased land degradation and pasture scarcity was attributed to increased overuse and mismanagement of the *free*-*for*-*all* communal fields in Chepareria. To fully exploit the land, individuals felt that they could better manage the vast lands if they were demarcated and boundaries established. Following the exit of the colonialists and the subsequent failure of the highly disliked group ranch management system, individuals seized this opportunity to wind up the group ranch management which was poorly coordinated in favour of individual landholdings; one which they had some degree of ownership, independence and control. Previous studies amongst enclosures owners in Somaliland by Gaani (2002) and in Ethiopia by Keene (2008) and McCarthy et al. (2003) have shown that individuals felt that they could better utilize and manage the land if they owned it. However, in some instances as indicated in research findings by Keene (2008), the allocation of grazing commons to individual private holders is also common when the state believes or assumes that privatization through individualization will encourage a more responsible use of the land. While the elements of individual willingness and government support for the establishment of enclosures in Chepareria are evident; the bottom line here lies in the realization that, by establishing enclosures, individuals in Chepareria not only have independence in land management and utilization but also gain the accruing land use/management benefits as observed by Saxer (2014) in Chepareria.

#### Crop production

The significance of farming as a factor for the establishment of enclosures reiterates previous findings by BurnSilver (2007) and Galvin (2009) in East African rangelands who observed that cultivation agriculture is gaining popularity and spread among East African pastoralists today. Consequently, pastoralists are cultivating where rain-fed or irrigated agriculture is a possibility. In Chepareria, two arguments can be made on the need to enclose land for farming. In the wetter areas of Chepareria, rain-fed agriculture is a major possibility as observed in the characterization of enclosure management systems in Chepareria by Wairore et al. (2015b). In these areas, market-oriented agriculture enables individuals to not only derive income but also produce diverse enclosure marketable products. In the lower altitude areas, agriculture is done on a subsistence basis. Second, previous studies on enclosures in Chepareria by Makokha et al. (1999), Wernersson (2013) and Karmebäck (2014) observed and reported that enclosures have reduced herding needs amongst enclosure owners in Chepareria hence individuals have more time for cultivation. These findings are consistent with those of Galvin et al. (2002) which describe that the increasing human population coupled with a relatively constant livestock population have encouraged individuals to diversify their income streams to make ends meet. Consequently, the need for cultivation/crop farming is not due to a decline in benefits derived from the livestock enterprise or the need to lease out land to outsiders perceived to have better farming skills as stated in previous studies by Hogg (1997), Gebre (2004) and Ayalew (2009).

#### Curbing land degradation

The successful rehabilitation of the most degraded areas in the demonstration plots set up in schools and churches made more individuals interested in enclosing their land as they associated enclosures with rangeland restoration. While rangeland enclosures were not specifically established to curb land degradation in Chepareria; enclosures have increased flexibility in the management of land use, fodder and livestock hence enabling households to not only eke a living, diversify sources of livelihood but also address land degradation in Chepareria. Our findings are similar to those of studies in Somalia which indicated that individuals still fence off most degraded areas within their own enclosures in order to protect them from indiscriminate use (Gaani 2002) while in Ethiopia, it is being done to curb land degradation (Forester 2002; WOCAT 2003; Nedessa et al. 2005; Napier and Desta 2011).

#### Diverse ecosystem services and environmental benefits

In Chepareria, the establishment of enclosures helped reduce communal use, regulate grazing and enhanced proper management of the enclosed areas which has fostered the recovery of formerly degraded lands. Increased vegetation cover has helped increase soil cover thus reducing losses of soil moisture through evapotranspiration. Increased soil cover has also been essential in facilitating improved water infiltration while reducing soil erosion. Increased litter deposition and carbon sequestration have also improved fertility hence increased productivity. Agroforestry practices have helped regulate the hydrological cycle, reduce wind and water soil erosion through their root binding action and increased rainfall induction. Previous studies have reported that enclosure owners benefit from various ecosystem services including improved water infiltration and retention, soil fertility, shade and erosion control (Wasonga et al. 2011; Mureithi et al. 2010; Svanlund 2014). In fact, previous studies in Ethiopia have reported that ecological change is a key reason for the establishment of enclosures (Keene 2008). As an integrated landscape approach, enclosures offer various environmental benefits such as soil stability, improved hydrological cycles, nutrients recharge and exchange and carbon sequestration on a landscape level (Scherr et al. 2012).

### Rangeland enclosure trade-offs: Have they shifted risks of land degradation from communal rangelands to private allotments?

While enclosures in Chepareria were not mainly established for land rehabilitation but to address pasture scarcity in Chepareria; the rapid ecological change witnessed within enclosed areas has proven that enclosures can be used as a management tool for the rehabilitation of degraded rangelands. Similar results have been reported by numerous previous studies in SSA (Mekuria et al. 2007; Mureithi et al. 2010; Verdoodt et al. 2010; Mekuria and Veldkamp 2012; Mekuria and Aynekulu 2013). Ecological restoration in the formerly degraded communal rangelands has been fostered by increased flexibility in land, fodder and livestock management in Chepareria as observed by Wairore et al. (2015b). While enclosures have been able to address land degradation, they have also reduced available communal land, increased land-based conflict within individual allotments, commoditized land, and created wealth stratification amongst households in Chepareria as observed in previous studies by Wairore et al. (2015a) in Chepareria. Ecologically, enclosures have significantly shifted risks of degradation from communal rangelands to private allotments by reducing available communal land hence restricting grazing to enclosed areas. Where grazing and intensive use of rangeland is not appropriately regulated; risks of land degradation within enclosed areas will be significantly high over time.

## Conclusion

Rangeland enclosures in Chepareria existed long before land management interventions by Vi-AF and were mainly established for boundary demarcation, alleviate pasture scarcity and foster proper land management in Chepareria. By increasing flexibility in land use, fodder and livestock management; households have been able to restore degraded areas over time and benefit from various ecosystem and environmental services. If the use and upscaling of rangeland enclosures is to be successful; technical interventions will have to be made to allow a more intensive use of rangeland resources. If this is not done, there are chances that land use fragmentation and management through rangeland enclosures will shift risks of degradation from previously communal rangelands to private allotments established through the enclosure movement.
